# Dynamic monitoring of neutrophil/lymphocyte ratio, APACHE II score, and SOFA score predict prognosis and drug resistance in patients with *Acinetobacter baumannii–calcoaceticus* complex bloodstream infection: a single-center retrospective study

**DOI:** 10.3389/fmicb.2024.1296059

**Published:** 2024-01-23

**Authors:** Zhiyong Wei, Lina Zhao, Jia Yan, Xuejie Wang, Qun Li, Yuanyuan Ji, Jie Liu, Yan Cui, Keliang Xie

**Affiliations:** ^1^Department of Critical Care Medicine, Tianjin Medical University General Hospital, Tianjin, China; ^2^Department of Pathogen Biology, School of Basic Medical Sciences, Tianjin Medical University, Tianjin, China; ^3^Department of Anesthesiology, Tianjin Institute of Anesthesiology, Tianjin Medical University General Hospital, Tianjin, China

**Keywords:** disease severity score, neutrophil/lymphocyte ratio (NLR), Carbapenem-resistant *Acinetobacter baumannii–calcoaceticus* complex (CRAB), risk factor, bloodstream infection (BSI), sepsis

## Abstract

**Objective:**

This study aimed to evaluate the clinical value of dynamic monitoring of neutrophil/lymphocyte ratio (NLR), APACHE II (Acute Physiology and Chronic Health Evaluation II) score, and Sequential Organ Failure Assessment (SOFA) score in predicting 28-day prognosis and drug resistance in patients with bloodstream infection with *Acinetobacter baumannii–calcoaceticus* complex (Abc complex).

**Patients and methods:**

In this research, individuals admitted to Tianjin Medical University General Hospital from January 2017 to March 2023 with bloodstream infections and a minimum of one Abc complex positive blood culture were chosen. The risk factors for the 28-day prognosis and drug resistance were analyzed using logistic regression. The NLR, APACHE II score, and SOFA score were evaluated for predicting 28-day prognosis and drug resistance using an ROC curve analysis. The data were analyzed using R Studio to find correlations and conduct survival analysis with the Kaplan–Meier method.

**Results:**

The final statistical analysis included a total of 129 patients with bloodstream infections caused by Abc complex. Independent risk factors predicting mortality within 28 days were identified as follows: the SOFA score and APACHE II scores at 24 h, and APACHE II scores at 72 h after the onset of blood infection (*p* < 0.05). NLR, SOFA score, and APACHE II score did not predict drug resistance. Patients with Carbapenem-resistant *Acinetobacter baumannii–calcoaceticus* complex (CRAB) had shorter survival times than those with carbapenem-sensitive strains (40.77 days vs. 47.65 days, respectively, *p* = 0.0032).

**Conclusion:**

The prognosis of Abc complex bloodstream infection is affected by both SOFA and APACHE II scores. Both scoring systems have similar prognostic values at different time points after infection, but for computational convenience, it is recommended to use the SOFA score. NLR exhibits limited effectiveness in predicting mortality within 28 days. Carbapenem-resistant individuals with Abc complex experience significantly reduced survival time. None of the three factors—SOFA score, APACHE II score, and NLR—can early predict the occurrence of CRAB infections effectively.

## Introduction

Clinically significant *Acinetobacter* species, such as *A. baumannii*, *A. nosocomialis*, *A. pittii*, *A. seifertii*, and *A. lacticae* (also called *A. dijkshoorniae*), as well as the environmental species *A. calcoaceticus*, are collectively designated members of the so-called *Acinetobacter baumannii–calcoaceticus* complex (Abc complex). Among the Abc complex species, *A. baumannii* is the most common cause of human infections (Castanheira et al., 2023). Abc complex has become one of the major pathogenic bacteria causing infection outbreaks in hospitals and is considered to be an influential cause of current nosocomial infection outbreaks in intensive care units worldwide (Qureshi et al., 2015). Abc complex demonstrated a pronounced propensity for elevated levels of drug resistance, leading to prolonged hospital stays and increased healthcare expenditures (Lee et al., 2022). Previous research has indicated that the mortality rate among individuals with bloodstream infections caused by Abc complex ranges from 11.3 to 66% (Nutman et al., 2014). Understanding the clinical characteristics and drug resistance of patients with Abc complex bloodstream infection is of great significance for identifying high-risk patients, reducing mortality, and improving prognosis.

Sepsis is defined as life-threatening organ dysfunction caused by a dysregulated host response to infection (Singer et al., 2016). The prevalence of intensive care unit-acquired sepsis caused by multidrug-resistant Abc complex infection is on the rise, and it can readily coexist with severe septicemia. In the pathogenesis of sepsis, an exaggerated systemic inflammatory response and compromised immune function play pivotal roles, with neutrophils and lymphocytes serving as essential cellular components. The systemic inflammatory responses contribute to an elevation in the proportion of neutrophils, while a reduction in lymphocyte counts indicates immune suppression in sepsis. Hence, the ratio of neutrophils to lymphocytes partially reflects the excessive inflammatory response and immune suppression within the body, thereby serving as a potential biomarker for assessing the severity of sepsis (Schroeder et al., 2001). In contrast to single neutrophils count (NEU) and lymphocyte count (LYM), NLR is able to reflect the dynamic equilibrium state of both and thus the interplay between inflammatory response and immune state of the body (Schroeder et al., 2001). The APACHE II score is widely recognized as the gold standard for assessing the severity of a critically ill patient, while the SOFA score provides an objective evaluation of a patient’s condition and boasts simplicity, reliability, and accessibility. Both scores hold immense value in evaluating the status, efficacy, and prognosis of patients with severe infections. It is imperative to conduct dynamic assessments of both APACHE II and SOFA scores throughout the treatment process for critically ill patients.

Earlier research has indicated a correlation between the incidence and outcome of Abc complex bacteremia with the APACHE II score and SOFA score (Liu et al., 2016; Park et al., 2018). Limited research has been conducted on the dynamic monitoring of the neutrophil/lymphocyte ratio, APACHE II score, and SOFA score in predicting drug resistance and 28-day prognosis among individuals with bloodstream infection caused by Abc complex. If these indicators can effectively predict Abc complex resistance and prognosis, it would significantly contribute to the management of nosocomial infections and reduction of mortality rates. This study aims to dynamically monitor variations in the neutrophil/lymphocyte ratio, APACHE II score, and SOFA score for accurate prediction of drug resistance and 28-day prognosis. Our objective is to enhance both survival rates and survival times among individuals affected by bloodstream infections associated with Abc complex.

## Materials and methods

### Hospital setting and study population

In this study, a cohort of 129 patients diagnosed with bloodstream infection caused by Abc complex and receiving treatment at Tianjin Medical University General Hospital in China between January 2017 and March 2023 were included as participants. The study protocol was approved by the Ethics Committee of the hospital (Approval No. IRB2022-[WZ-077]). Demographic information of patients, clinical markers, biochemistry results, pre-existing medical conditions (such as diabetes, high blood pressure, heart disease, renal insufficiency, and malignant tumors), invasive procedures (including blood purification, surgery, mechanical ventilation, deep vein catheterization, and fiberoptic bronchoscopy), ICU admission, total length of stay, and other indicators were collected. Inclusion criteria were as follows: (1) Adults aged 18 or above with complete and accessible data for analysis. (2) Positive Abc complex test in peripheral blood on one or more occasions. (3) Hospital stay of at least 72 h. Exclusion criteria were as follows: (1) Discharged within 72 h (*n* = 4). (2) Solid tumor progression or hemopathy (*n* = 5). (3) Previous use of immunosuppressants, cytotoxic drugs, or hormones (*n* = 5). The NLR was calculated using the absolute neutrophil/lymphocyte ratio at admission and 24, 48, and 72 h after diagnosis of bloodstream infection with *A. baumannii*, based on routine peripheral blood tests. The APACHE II and SOFA scores were derived from clinical data and laboratory test results at admission and 24, 48, and 72 h after diagnosis of bloodstream infection with Abc complex. Diagnostic criteria for blood flow infection of Abc complex: the patient developed obvious systemic inflammatory response syndrome (SIRS) 48 h after admission, and was diagnosed as nosocomial infection of Abc complex bloodstream infection when it was isolated from one or more blood cultures (El-Shazly et al., 2015). The initiation of Abc complex bacteremia was determined based on the date when the initial blood culture yielded positive results, and the duration of survival was measured from the onset of bacteremia. All individuals were monitored for a duration of 28 days, considering the 28-day result as the final assessment point, and tallying instances of both survival and mortality. CRAB is characterized by exhibiting resistance to at least one of the following drugs: ertapenem, meropenem, or imipenem in drug sensitivity tests (Maragakis and Perl, 2008). The patients were categorized into two groups based on their 28-day prognosis: the survival group (*n* = 66) and the non-survival group (*n* = 63). They were divided into two groups based on drug sensitivity: CRAB group (*n* = 92) and carbapenem-sensitive *Acinetobacter baumannii–calcoaceticus* complex (CSAB) group (*n* = 37). The data of the two groups were statistically analyzed. Sepsis was diagnosed based on the criteria outlined in Sepsis 3.0 (Singer et al., 2016).

Blood culture is considered the gold standard for diagnosing bloodstream infections (BSI). In this study, we conducted blood cultures on patients presenting symptoms indicative of infection, such as fever, cold, chills, and hypotension. The blood of infected patients was cultured using the BD Bactec TM FX400 blood culture instrument. The drug sensitivity and identification of *Acinetobacter baumannii–calcoaceticus* complex were performed by the French Merieux VITEK−2 Compact and VITEK MS automatic microbial analyzer, respectively. The interpretation of antimicrobial susceptibility tests followed the Clinical and Laboratory Standards Institute (CLSI) guidelines (Dalyan Cilo and Ener, 2021). For outcomes that were not covered by CLSI, the recommendations provided by the European Committee on Antimicrobial Susceptibility Testing (EUCAST) were consulted (Giske et al., 2022). WHONET 5.5 software provided by the WHO Drug Resistance Surveillance Agency was used to analyze the strain resistance. The situation of CRAB bloodstream infection was monitored and its risk factors, prognosis, and drug resistance were analyzed.

### Statistical analysis

Relevant data was processed by employing the statistical software SPSS 26.0. For normally distributed measurements with homogeneous variance, we presented them as mean ± standard deviation ( $\bar{x}$ ± S) and employed a two independent samples *t*-test for analysis. In cases where measurements had non-normal distributions or uneven variances, we represented them using median (quartile) [M (QL, QU)] values, employing a rank-sum test for group comparisons instead. Categorical variables were expressed through case numbers (constituent ratios), and analyzed using χ^2^ tests between groups. Both univariate and multivariate analyzes were conducted to identify risk factors for mortality in bacteremia patients. Additionally, R Studio language-based statistical software aided in determining which factors played a dominant role in predicting outcomes for *A. baumannii* bacteremia patients. The survival analysis employed the Kaplan–Meier method, while the Log-rank test was utilized to compare differences between the CRAB and CSAB groups, as well as between sepsis and non-sepsis groups. To determine the optimal cut-off point for predicting death, we plotted receiver operator characteristic curves (ROC curves) for the APACHE II score and SOFA score. Sensitivity, specificity, and cut-off values were calculated for each ROC analysis. The optimal cut-off value was identified based on achieving the highest sensitivity and specificity. We also computed the Youden index (sensitivity + specificity −1) to determine this optimal threshold value. All statistical tests were conducted as two-tailed tests, with a significance level set at *p* < 0.05.

## Results

### General condition of patients

**Figure 1:**
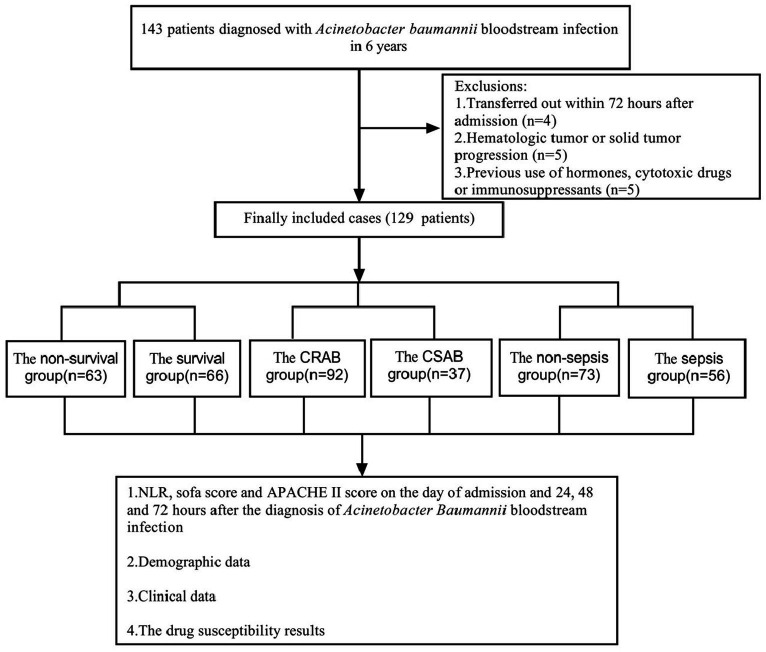
Flow chart for patient selection. A total of 143 *A. baumannii* blood culture positive specimens were analyzed for drug resistance and the clinical data. After excluding 14 patients, 129 cases of *A. baumannii* bacteremia remainder were included in the clinical data analysis.

This study extended the results of studies completed between February 2017 and March 2022, with the same inclusion criteria, and all patients were blood culture-positive for Abc complex (Wei et al., 2022). In this study, we used information from 110 patients collected in the previous study and added 19 additional patients. During the period from February 2017 to March 2023, a comprehensive collection of 143 individuals diagnosed with bloodstream infection caused by Abc complex was obtained, 4 patients with less than 72 h of hospital stay, 5 patients with hematologic malignancies who could not calculate NLR, and 5 patients with previous use of hormones, cytotoxic drugs or immunosuppressive agents were excluded, and 129 patients were finally included for statistical analysis (Figure 1). There were 85 men (65.89%) and 44 women (34.11%). The age of the participants varied between 28 and 94 years, with an average age of 64.83 ± 14.78 years. The 129 cases of *A. baumannii* were mainly from the general ICU (56 cases), general surgery department (17 cases), emergency ICU (15 cases), neurology department (8 cases), hematology department (5 cases), etc. Patients isolated from the ICU with bloodstream infections of Abc complex had significantly higher rates of drug resistance than those in other units, and the site of infection was primarily from a lower respiratory tract infection.

### Drug sensitivity results for Abc complex bacteremia

**Figure 2:**
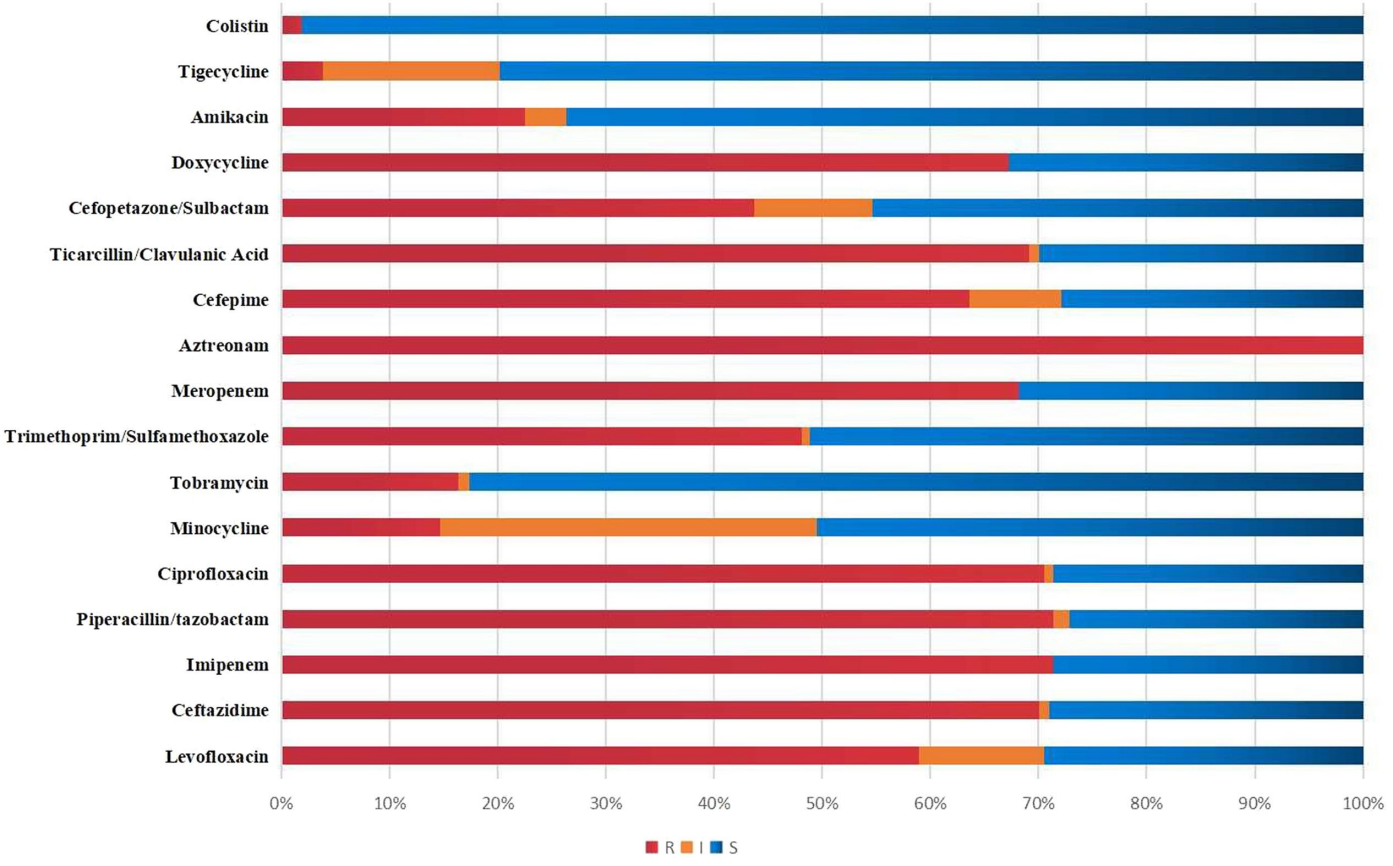
*In vitro* susceptibility results of 129 patients with *A. baumannii* bacteremia.

Figure 2 visually presents the results of antimicrobial susceptibility tests conducted in a lab. The resistance rates for Meropenem, Imipenem, Levofloxacin, Cefoperazone/sulbactam, and Amikacin were 68.22, 71.32, 58.91, 43.70, and 22.48%, respectively. Tigecycline and colistin had resistance rates of only 3.88 and 1.87%.

### Data on Abc complex bacteremia patients

Of all the patients (*n* = 129) in this study, 63 died within 28 days, resulting in a mortality rate of approximately 48.84%. No significant variations were found in terms of age, gender, NLR at admission, and the prevalence of underlying conditions such as hypertension, diabetes, coronary heart disease, and malignant tumors between the group that survived and the group that did not survive (all *p* > 0.05). Compared to the group of patients who survived, the non-survival group had a higher percentage of individuals admitted to the ICU, a longer overall hospital stay, and a greater proportion of invasive procedures, such as hemopurification therapy, deep vein catheterization, and fiberbronchoscopy. Additionally, the non-survival group had a lower percentage of individuals undergoing surgery compared to those in the survival group (all *p* < 0.05). The incidence rates of sepsis and CRAB were significantly elevated in the non-survival group when compared to the survival group (p < 0.05). The admission, bloodstream infection day, 48-h and 72-h SOFA scores, and APACHE II scores were significantly higher in the non-survival group than the survival group (all p < 0.05). The NLR of the non-survival group on the day of bloodstream infection, as well as at 48 and 72 h after infection, was significantly elevated compared to that of the survival group (all *p* < 0.05) (Table 1). According to the different time points of the score, we built three models. In Model 1 of the study on the 24-h NLR, SOFA, and APACHE II scores, it was found that both the 24-h SOFA score and APACHE II score have significant implications for mortality within 28 days. Specifically, for every one standard deviation increase in the 24-h SOFA score, there is a 40.8% increased risk of death (1.408, 95% CI:1.025 to 1.932). Similarly, for every one standard deviation increase in the 24-h APACHE II score, there is a 13.3% increased risk of death within 28 days (1.133, 95% CI:1.028 to 1.250) (all *p* < 0.05). When including 48 h NLR, SOFA, and APACHE II scores in Model 2, it was determined that these three scores did not have a significant impact on mortality at 28 days. However, after incorporating the 72 h NLR, SOFA, and APACHE II scores into Model 3, we discovered that for every one standard deviation increase in the 72 h APACHE II score, there was a significant 13.9% rise in mortality at 28 days (1.139, 95% CI,1.011 to 1.284). The three models consisting of the 24 h SOFA score as well as the 24 h and 72 h APACHE II scores were found to possess substantial predictive value for determining the likelihood of mortality within a span of 28 days (Table 2).

**Table 1:** Univariate analysis of the risk factors predicting 28-day death in patients with *A. baumannii* bacteremia.

	Survival (*n* = 66)	Non-survival (*n* = 63)	*χ*^2^/*t*/*z* value	*p*-value
Age (mean ± SD)/years	61.45 ± 15.96	68.37 ± 12.62	−2.719^a^	0.061
Male [*n* (%)]	42/66 (63.64)	43/63 (68.25)	0.037^b^	0.847
ICU stay [*n* (%)]	47/66 (71.21)	58/63 (92.06)	9.254^b^	0.002
Hospital stay [d,M (Ql, Qu)]	40.50 (20.00, 67.00)	24.00 (10.00, 41.00)	−3.372^c^	0.001
Complications [cases (%)]				
Hypertension [*n* (%)]	36/66 (54.55)	32/63 (50.80)	0.182^b^	0.670
Diabetes [*n* (%)]	16/66 (24.24)	18/63 (28.57)	0.311^b^	0.577
Coronary heart disease [*n* (%)]	21/66 (31.82)	26/63 (41.27)	1.243^b^	0.265
Renal insufficiency [*n* (%)]	13/66 (19.70)	40/63 (63.49)	25.541^b^	0.000
Malignant tumor [*n* (%)]	19/66 (28.79)	28/63 (44.44)	3.412^b^	0.065
CRRT [*n* (%)]	6/66 (9.09)	22/63 (34.92)	12.654^b^	0.000
Surgery [*n* (%)]	45/66 (68.18)	13/63 (20.63)	29.446^b^	0.000
Mechanical ventilation [*n* (%)]	45/66 (68.18)	54/63 (85.71)	5.551^b^	0.018
Deep vein catheterization [*n* (%)]	39/66 (59.09)	60/63 (95.24)	23.598^b^	0.000
Bronchofibroscope [*n* (%)]	19/66 (28.79)	51/63 (80.95)	34.537^b^	0.000
0 h NLR [M (Ql, Qu)]	5.41 (3.15, 16.35)	8.88 (3.50, 21.16)	−0.966^c^	0.334
24 h NLR [M (Ql, Qu)]	10.46 (4.68, 22.04)	20.48 (7.53, 33.00)	−2.773^c^	0.006
48 h NLR [M (Ql, Qu)]	10.08 (4.60, 18.40)	25.14 (10.39, 42.00)	−4.448^c^	0.000
72 h NLR [M (Ql, Qu)]	5.90 (4.18, 12.68)	23.50 (7.87, 40.50)	−4.356^c^	0.000
0 h SOFA score [M (Ql, Qu)]	3.00 (2.00, 6.00)	5.00 (3.00, 8.00)	−2.773^c^	0.006
24 h SOFA score [M (Ql, Qu)]	5.00 (3.00, 6.00)	10.00 (8.00, 13.00)	−8.038^c^	0.000
48 h SOFA score [M (Ql, Qu)]	4.00 (2.75, 6.00)	12.00 (8.00, 14.00)	−8.038^c^	0.000
72 h SOFA score [M (Ql, Qu)]	4.00 (2.00, 6.00)	13.00 (9.00, 15.00)	−8.313^c^	0.000
0 h APACHE II score [M (Ql, Qu)]	12.00 (10.00, 18.25)	17.00 (12.00, 24.00)	−3.174^c^	0.002
24 h APACHE II score [M (Ql, Qu)]	14.00 (10.75, 19.00)	30.00 (24.00, 33.00)	−7.822^c^	0.000
48 h APACHE II score [M (Ql, Qu)]	13.00 (10.00, 22.00)	28.00 (23.00, 34.00)	−7.617^c^	0.000
72 h APACHE II score [M (Ql, Qu)]	12.00 (10.00, 20.00)	31.00 (26.00, 35.00)	−8.226^c^	0.000
CRAB [*n* (%)]	39/66 (59.10)	53/63 (84.13)	9.877^b^	0.002
Sepsis [*n* (%)]	13/66 (19.70)	43/63 (68.25)	30.936^b^	0.000

ICU, intensive care unit; NLR, neutrophil/lymphocyte ratio; SOFA, Sequential Organ Failure Assessment score; APACHE II, Acute Physiology and Chronic Health Evaluation II score; CRAB, carbapenem-resistant A. baumannii; CRRT, continuous renal replacement therapy. *t*, Student t-test; *χ*^2^, Chi-square test; z, Rank-sum test. *p*-value, <0.05 was considered as statistically significant. a for *t*, b for *χ*^2^, and c for *z*.

**Table 2:** Multivariate analysis of the different time points of NLR, APACHE II score, and SOFA score predicting 28-day death in patients with *A. baumannii* bacteremia.

	OR	95% CI	*p*-value
Model 1			
24 h NLR	1.000	0.996 ~ 1.003	0.897
24 h SOFA score	1.408	1.025 ~ 1.932	0.034
24 h APACHE II score	1.133	1.028 ~ 1.250	0.012
Model 2			
48 h NLR	1.038	0.995 ~ 1.083	0.084
48 h SOFA score	1.215	0.948 ~ 1.557	0.124
48 h APACHE II score	1.139	0.974 ~ 1.332	0.103
Model 3			
72 h NLR	1.009	0.973 ~ 1.046	0.627
72 h SOFA score	1.253	0.968 ~ 1.621	0.087
72 h APACHE II score	1.139	1.011 ~ 1.284	0.033

Model 1: ICU stay, hospital stay, renal insufficiency, CRRT, surgery, deep vein catheterization, mechanical ventilation, bronchofibroscope, CRAB, sepsis, NLR 24 h, SOFA 24 h, and APACHE II 24 h.

Model 2: ICU stay, hospital stay, renal insufficiency, CRRT, sur gery, deep vein catheterization, mechanical ventilation, bronchofibroscope, CRAB, sepsis, NLR 48 h, SOFA 48 h, and APACHE II 48 h.

Model 3: ICU stay, hospital stay, renal insufficiency, CRRT, surgery, deep vein catheterization, mechanical ventilation, bronchofibroscope, CRAB, sepsis, NLR 72 h, SOFA 72 h, and APACHE II 72 h.

ICU, intensive care unit; NLR, neutrophil/lymphocyte ratio; SOFA, Sequential Organ Failure Assessment score; APACHE II, Acute Physiology and Chronic Health Evaluation II score; CRAB, carbapenem-resistant *A. baumannii*; CRRT, continuous renal replacement therapy.

There were no statistically significant differences observed in terms of gender, age, NLR, SOFA, and APACHE II scores upon admission, as well as the prevalence of underlying conditions such as hypertension, diabetes, coronary heart disease, malignant tumor, and renal insufficiency between the CRAB group (*n* = 92) and CSAB group (*n* = 37) (all *p* > 0.05). However, compared to the CSAB group, a higher proportion of patients in the CRAB group were admitted to the ICU and required mechanical ventilation or deep venous catheterization (all *p* < 0.05), while a lower proportion underwent surgery. Notably, mortality rates and sepsis incidence were significantly higher in the CRAB group than in the CSAB group (*p* < 0.05). Furthermore, on the day of bloodstream infection and at 24 h, 48 h, and 72 h post-infection, NLR values along with SOFA score and APACHE II score were all significantly elevated in the CRAB group compared to those in the CSAB group (all *p* < 0.05). Multivariate logistic regression analysis revealed that only ICU admission independently contributed to an increased risk for CRAB development (Table 3).

**Table 3:** Univariate and multivariate analyzes for predicting drug resistance in patients with *A. baumannii* bacteremia.

	CRAB (*n* = 92)	CSAB (*n* = 37)	*χ*^2^/t/z value	*p*-value	Multivariate analysis			
					RR	95.0% CI		*p* value
						Lower	Upper	
Age [years, M (Ql, Qu)]	66.00 (56.25, 74.00)	68.00 (58.50, 75.00)	−0.901^c^	0.367				
Male [*n* (%)]	61 (66.30)	26 (70.27)	0.189^b^	0.66				
ICU stay [*n* (%)]	84/92 (91.30)	21/37 (56.76)	20.798^b^	0.000	0.130	0.030	0.561	<0.005
Hospital stay [d, M (Ql, Qu)]	28.50 (15.00, 47.75)	40.00 (16.50, 71.00)	0.731^c^	0.001	0.995	0.985	1.005	0.349
0 h NLR [M (Ql, Qu)]	8.81 (3.66, 17.45)	4.39 (3.04, 12.75)	−1.862^c^	0.063				
24 h NLR [M (Ql, Qu)]	15.78 (6.91, 32.49)	10.41 (4.23, 19.25)	−2.580^c^	0.010	0.998	0.993	1.003	0.481
48 h NLR [M (Ql, Qu)]	18.83 (7.91, 35.90)	7.60 (4.35, 17.37)	−3.369^c^	0.001	1.055	0.984	1.130	0.131
72 h NLR [M (Ql, Qu)]	18.10 (5.22, 39.07)	5.60 (3.24, 16.70)	−3.544^c^	0.000	0.999	0.951	1.050	0.980
0 h SOFA score [M (Ql, Qu)]	5.00 (2.00, 7.00)	3.00 (2.00, 6.00)	−1.540^c^	0.123				
24 h SOFA score [M (Ql, Qu)]	8.00 (5.00, 12.00)	5.00 (4.00, 7.00)	−3.395^c^	0.001	1.184	0.840	1.669	0.336
48 h SOFA score [M (Ql, Qu)]	8.00 (5.00, 12.00)	4.00 (3.00, 6.00)	−3.574^c^	0.000	0.922	0.537	1.584	0.769
72 h SOFA score [M (Ql, Qu)]	9.00 (4.00, 14.00)	3.00 (2.50, 6.00)	−3.837^c^	0.000	1.140	0.735	1.769	0.558
0 h APACHE II score [M (Ql, Qu)]	16.00 (11.00, 23.00)	13.00 (10.00, 24.00)	−0.696^c^	0.486				
24 h APACHE II score [M (Ql, Qu)]	24.00 (14.00, 31.00)	15.00 (11.00, 24.00)	−2.858^c^	0.004	0.988	0.896	1.089	0.808
48 h APACHE II score [M (Ql, Qu)]	24.00 (15.00, 30.00)	14.00 (11.00, 23.00)	−3.004^c^	0.003	0.879	0.720	1.073	0.205
72 h APACHE II score [M (Ql, Qu)]	25.00 (15.00,32.00)	12.00 (10.00, 23.00)	−3.335^c^	0.001	1.092	0.918	1.299	0.319
Complications [cases (%)]								
Hypertension [*n* (%)]	44/92 (47.83)	17/37 (45.95)	0.037^b^	0.847				
Diabetes [*n* (%)]	22/92 (23.91)	12/37 (32.43)	0.987^b^	0.321				
Coronary heart disease [*n* (%)]	59/92 (64.13)	23/37 (62.16)	0.044^b^	0.834				
Renal insufficiency [*n* (%)]	39/92 (42.39)	14/37 (37.84)	0.226^b^	0.634				
Malignant tumor [*n* (%)]	34/92 (36.96)	13/37 (35.14)	0.038^b^	0.846				
CRRT [*n* (%)]	21/92 (22.83)	7/37 (18.92)	0.237^b^	0.626				
Surgery [*n* (%)]	39/92 (42.39)	19/37 (51.35)	0.856^b^	0.355				
Mechanical ventilation [*n* (%)]	77/92 (83.70)	22/37 (59.46)	8.685^b^	0.003	0.604	0.156	2.341	0.466
Deep vein catheterization [*n* (%)]	76/92 (82.61)	23/37 (62.16)	0.237^b^	0.626				
Bronchofibroscope [*n* (%)]	57/92 (61.96)	13/37 (35.14)	8.029^b^	0.005	0.849	0.225	3.204	0.809
Survival [*n* (%)]	39/92 (42.39)	27/37 (72.97)	9.877^b^	0.002	2.076	0.341	12.618	0.428
Sepsis [*n* (%)]	45/92 (48.91)	11/37 (29.73)	3.953^b^	0.047	2.163	0.559	8.368	0.264

ICU, intensive care unit; NLR, neutrophil/lymphocyte ratio; SOFA, Sequential Organ Failure Assessment score; APACHE II, Acute Physiology and Chronic Health Evaluation II score; CRAB, carbapenem-resistant *Acinetobacter baumannii*; CSAB, carbapenem sensitive *Acinetobacter baumannii*; CRRT, continuous renal replacement therapy. *t*, Student *t*-test; *χ*^2^, Chi-square test; z, Rank-sum test; *p*-value, <0.05 was considered as statistically significant. a for *t*, b for *χ*^2^, and c for *z*.

**Figure 3:**
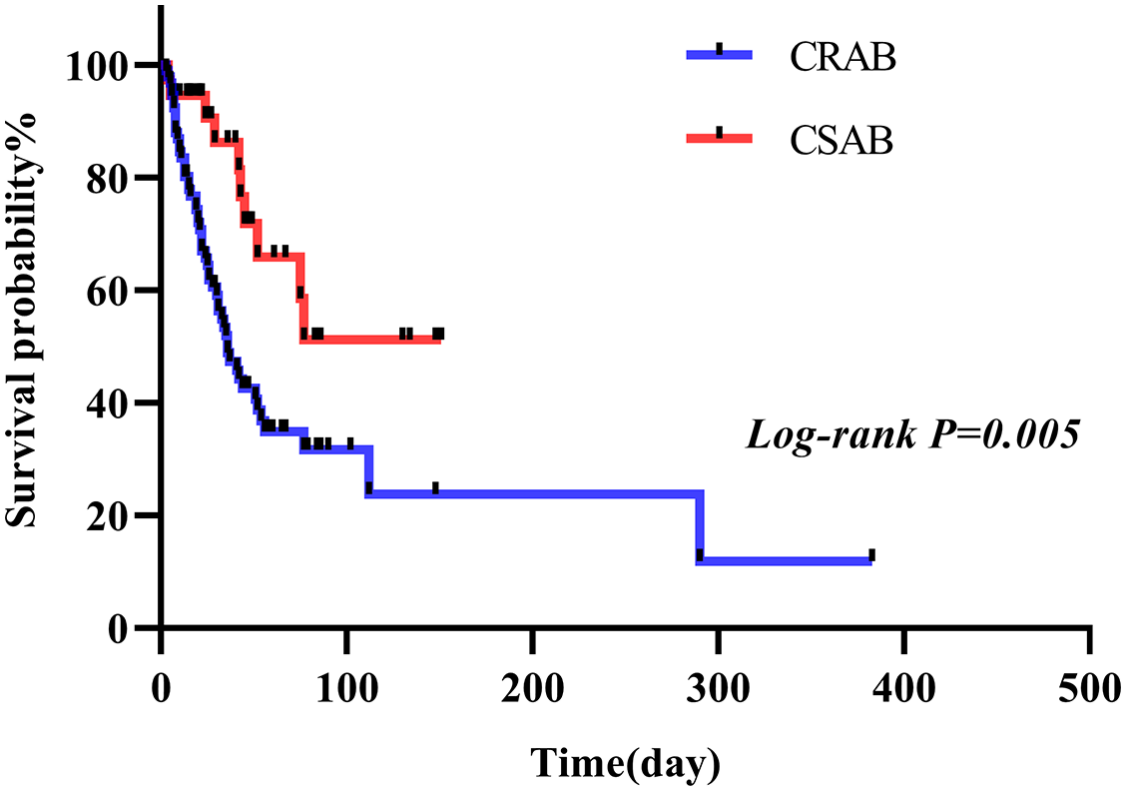
CRAB group and CSAB group survival curves of patients infected with *A. baumannii* bacteremia.

**Figure 4:**
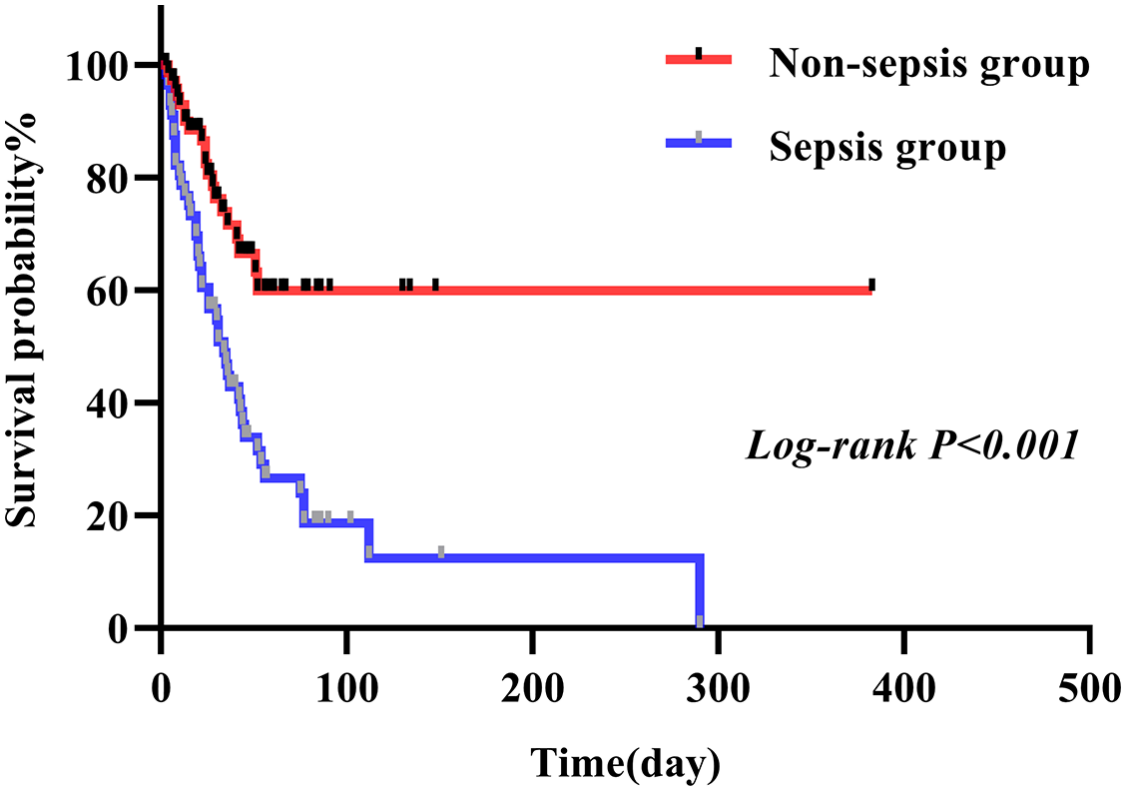
Sepsis and non-sepsis survival curves of patients infected with *A. baumannii* bacteremia.

**Figure 5:**
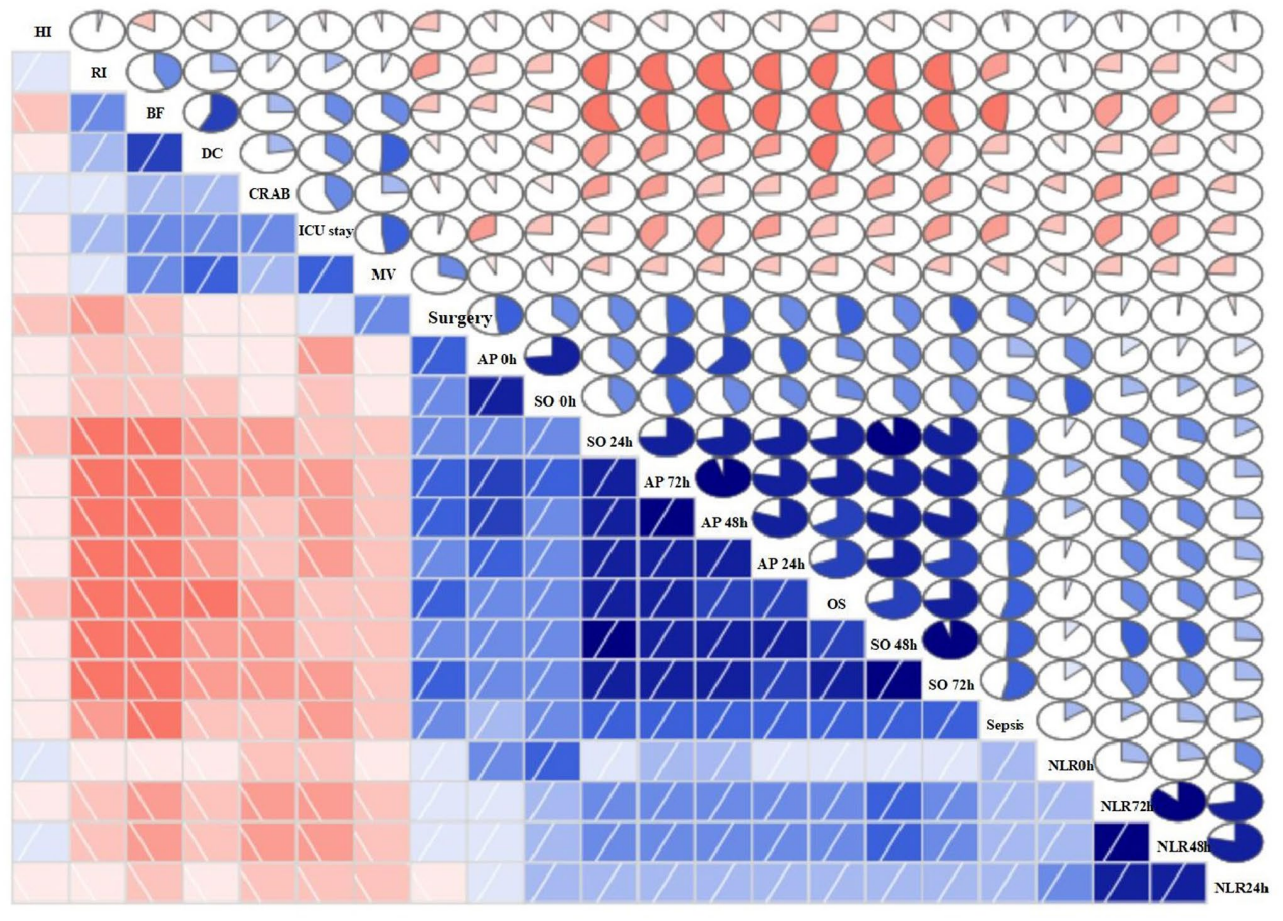
Correlogram of my data intercorrelations. HS, hospital stay; AP, APACHE II; SO, SOFA; CRAB, carbapenem-resistant *A. baumannii*; BF, bronchofibroscope; RI, renal insufficiency; DC, deep vein catheterization; OS, overall survival; MV, mechanical ventilation; NLR, neutrophil/lymphocyte ratio.

In this study, we conducted a comparison between the survival durations of patients with CRAB and CSAB bacteremia. Within the CRAB group, there were 92 cases observed, out of which 53 resulted in deaths. The survival curve exhibited a sharp decline. On the other hand, the CSAB group consisted of 37 cases with 10 deaths recorded, showing a relatively gradual decrease in the survival curve. By applying a log-rank test, it was determined that patients with CRAB had significantly shorter survival times compared to those with CSAB (*p* = 0.005) (Figure 3). At the same time, we conducted a comparison between the survival durations of patients with sepsis and those with non-septic Abc complex bacteremia. The average survival duration for sepsis patients was (41.52 ± 45.66) days, while it was (43.68 ± 51.89) days for non-sepsis patients. Among the 56 patients who developed sepsis, 43 of them passed away, resulting in a sharp decline in the survival curve. On the other hand, out of the 73 patients without sepsis, only 20 died, leading to a relatively gradual decrease in their survival curve. It is worth noting that the sepsis patients had a shorter survival duration compared to their non-septic counterparts (*p* < 0.001), as depicted in Figure 4. Utilizing R Studio statistical software for data analysis revealed that both 72 h APACHE II (R = 0.74) and 24 h SOFA (R = 0.73) exhibited strong correlations with patient prognosis (Figure 5).

### NLR, SOFA, and APACHE II scores can predict outcomes in Abc complex bacteremia

**Figure 6:**
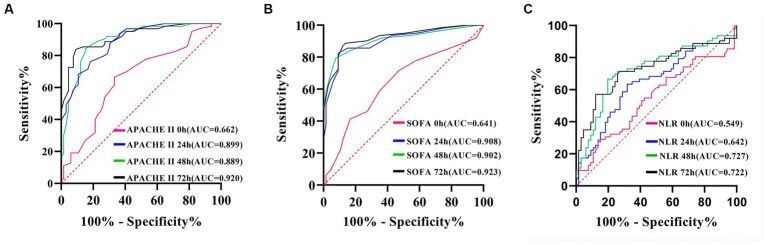
ROC curve of APACHE II score **(A)**, SOFA score **(B)** and NLR **(C)**, predicting 28-day prognosis of patients with *A. baumannii* bloodstream infection at each time point.

The maximum area under the curve (AUC) for APACHE II (Figure 6A) and SOFA scores (Figure 6B) was achieved at the 72-h mark. Using a cut-off value of 72-h SOFA >6, we predicted death at 28 days with a sensitivity of 88.89% and specificity of 86.36%. Similarly, using an APACHE II score > 23 at the same time point, we predicted death at 28 days with a sensitivity of 84.13% and specificity of 90.91%. The AUC for neutrophil-to-lymphocyte ratio (NLR) was highest at the 48-h mark (Figure 6C), where NLR > 20.8 served as the cut-off to predict death at 28 days, yielding a sensitivity of 57.14% and specificity of 87.88% (Table 4).

**Table 4:** Prediction value of NLR, SOFA, and APACHE II for 28-day prognosis of patients with *A. baumannii* bloodstream infection at each time point.

	Best cut-off value	AUC	95% CI	*p-*value	Specificity %	Sensitivity%
0 h NLR	19.7	0.549	0.448–0.650	0.338	84.85	28.57
24 h NLR	13.99	0.642	0.545–0.738	0.004	68.18	63.49
48 h NLR	20.8	0.727	0.636–0.818	0.000	87.88	57.14
72 h NLR	16.73	0.722	0.631–0.814	0.000	80.30	66.67
0 h SOFA	6	0.641	0.552–0.723	0.0043	83.33	41.27
24 h SOFA score	7	0.908	0.845–0.952	<0.0001	92.42	79.37
48 h SOFA score	7	0.902	0.837–0.947	<0.0001	90.91	82.54
72 h SOFA score	6	0.923	0.863–0.963	<0.0001	86.36	88.89
0 h APACHE II score	14	0.662	0.573–0.743	0.0008	66.67	66.67
24 h APACHE II score	21	0.899	0.834–0.945	<0.0001	84.85	84.13
48 h APACHE II score	22	0.889	0.821–0.937	<0.0001	81.82	76.19
72 h APACHE II score	23	0.920	0.858–0.960	<0.0001	90.91	84.13

NLR, neutrophil/lymphocyte ratio; SOFA, Sequential Organ Failure Assessment score; APACHE II, Acute Physiology and Chronic Health Evaluation II score.

### NLR, SOFA, and APACHE II scores had limited efficacy in predicting drug resistance

**Figure 7:**
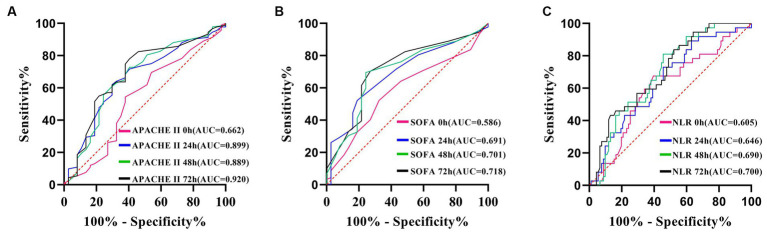
ROC curve of APACHE II score **(A)**, SOFA score **(B)** and NLR **(C)**, predicting drug resistance in patients with *A. baumannii* bloodstream infection at each time point.

The AUC for the APACHE II score (Figure 7A), the SOFA score (Figure 7B), and the NLR all reach their maximum at 72 h (Figure 7C). APACHE II > 14 was used as the cut-off point to predict the resistance of the Abc complex; the sensitivity was 78.26% and the specificity was 59.46%. With NLR > 19.76 as the cut-off point to predict the resistance of *the* Abc complex, the sensitivity was 47.83% and the specificity was 83.78%. The cut-off point of SOFA >5 was employed to predict the resistance of the Abc complex, resulting in a sensitivity rate of 70.65% and a specificity rate of 72.97% (Table 5).

**Table 5:** Prediction value of NLR, SOFA, and APACHE II on drug resistance in patients with *A. baumannii* bloodstream infection at each time point.

	Best cut-off value	AUC	95% CI	*p*-value	Specificity %	Sensitivity%
0 h NLR	5.57	0.605	0.515–0.690	0.0568	67.57	60.87
24 h NLR	14.02	0.646	0.557–0.728	0.0054	72.97	54.35
48 h NLR	18.33	0.690	0.603–0.769	0.0001	81.08	54.35
72 h NLR	19.76	0.700	0.613–0.777	<0.0001	83.78	47.83
0 h SOFA	4	0.586	0.496–0.672	0.1047	67.57	52.17
24 h SOFA score	7	0.691	0.603–0.769	0.0001	81.08	52.17
48 h SOFA score	5	0.701	0.614–0.778	0.0001	75.68	69.57
72 h SOFA score	5	0.718	0.632–0.793	<0.0001	72.97	70.65
0 h APACHE II score	14	0.539	0.449–0.627	0.5109	62.16	54.35
24 h APACHE II score	20	0.661	0.573–0.742	0.0026	70.27	61.96
48 h APACHE II score	16	0.669	0.581–0.750	0.0023	59.46	72.83
72 h APACHE II score	14	0.690	0.603–0.769	0.0004	59.46	78.26

NLR, neutrophil/lymphocyte ratio; SOFA, Sequential Organ Failure Assessment score; APACHE II, Acute Physiology and Chronic Health Evaluation II score.

## Discussion

Abc complex is the most commonly identified species in the genus *Acinetobacter* and it accounts for a large percentage of nosocomial infections, including bacteremia, pneumonia, and infections of the skin and urinary tract. A few key clones of *A. baumannii–calcoaceticus* are currently responsible for the dissemination of these organisms worldwide. Unfortunately, multidrug resistance is a common trait among these clones due to their unrivaled adaptive nature (Castanheira et al., 2023). While numerous studies have established a correlation between sepsis and NLR, APACHE II score, and SOFA score, limited research has been conducted on the predictive value of dynamically monitoring these factors for drug resistance in the Abc complex. Previously, we only studied the effects of SOFA and APACHE II scores 24 h after Abc complex bloodstream infection on prognosis, without conducting dynamic monitoring. This study further improved our previous study and increased the sample size to 129 cases. All 129 patients included were those who developed a bloodstream infection of Abc complex. The majority of the non-survivors (*n* = 63) were hospitalized patients in the ICU, and the 28-day fatality rate was 48.84%. The admission APACHE II score and SOFA score were higher in the non-survival group compared to the survival group, while the incidence of sepsis and CRAB was significantly elevated in the non-survival group (*p* < 0.05). The SOFA and APACHE II scores at 24, 48, and 72 h post-Abc complex bloodstream infection exhibit high specificity in predicting the prognosis of patients with Abc complex bloodstream infection within a 28-day period. The 72-h SOFA score demonstrates a high level of sensitivity, with an area under the ROC curve reaching up to 0.923. In our study, patients with Abc complex bloodstream infection were divided into two groups: CRAB (*n* = 92) and CSAB (*n* = 37). The findings indicated that patients in the CRAB group showed significantly elevated NLR, APACHE II, and SOFA scores at 24, 48, and 72 h compared to those in the CSAB group. Additionally, a higher incidence of ICU admission, mortality rate, and sepsis was observed among individuals in the CRAB group as opposed to the CSAB group. These findings suggest a correlation between the aforementioned factors and the development of bloodstream infections caused by CRAB. Moreover, patients in the CRAB group exhibited significantly lower survival rates compared to their counterparts in the CSAB group. However, our multivariate logistic regression analysis identified admission to ICU as an independent risk factor for CRAB infection. Additionally, we observed a correlation between invasive procedures and increased mortality rates and incidence of CRAB infection among patients. Among all enrolled patients, sepsis occurred in 56 cases—predominantly affecting males over 65 years old. Furthermore, we noted that septic patients had shorter survival times than those without sepsis.

- *Acinetobacter* accounted for 7.5% of bloodstream infection pathogens, after coagulase-negative *Staphylococcus*, methicillin-resistant *Staphylococcus aureus* (MRSA), *Pseudomonas aeruginosa,* and *Enterococcus* (Orsi et al., 2012). Potential factors for Abc complex infection include prolonged hospital stay, ICU admission, mechanical ventilation, invasive procedures, antibiotic exposure, and severe underlying diseases (Munoz-Price and Weinstein, 2008), with a fatality rate of 11.3% ~ 22.1% (Chen et al., 2005; Shete et al., 2009). The ICU has emerged as a high-risk setting for nosocomial infections due to the critical condition of patients and the multiple invasive interventions that result in compromised physical function, diminished immune capacity, and heightened susceptibility to external pathogens (Schaal et al., 2020). As the isolation rate of CRAB increases, the associated nosocomial infections pose a significant threat to public health (Bush and Jacoby, 2010). *Acinetobacter*’s primary mechanisms of resistance to carbapenem antibiotics encompass the production of β-lactamases (primarily class B and D), alteration of drug targets, modification of outer membrane permeability, and overexpression of efflux pumps. Among these factors, the primary determinant of drug resistance in the Abc complex is attributed to the intricate mechanism underlying enzyme production, with particular emphasis on the pivotal role played by the expression of the OXA gene (Poirel et al., 2000). In this study, differences were observed in the use of mechanical ventilation, fiberbronchoscopy, admission to the intensive care unit (ICU), NLR, SOFA score, and APACHE II score after infection between patients in the CRAB and CSAB groups through univariate logistic regression analysis. These findings suggest a potential association between these individual factors and the development of bloodstream infection caused by CRAB. The independent risk factor for CRAB was identified as ICU admission through the implementation of multivariate logistic analysis. The prevalence of bloodstream infection and drug resistance caused by Abc complex was notably elevated among ICU patients compared to those in other departments. This can be attributed to the critical condition of ICU patients and the use of antibacterial drugs with a broad spectrum, prolonged duration, and excessive dose, which further induce the production of CRAB. In this investigation, the utilization of mechanical ventilation was identified as a potential risk factor for CRAB bacteremia, aligning with findings from several prior studies (García-Garmendia et al., 2001). This was a logical conclusion, considering the prior findings indicating that respiratory failure enhances the colonization of Abc complex in the respiratory system, and such colonization poses a risk for developing Abc complex bacteremia (Jang et al., 2009). Respiratory insufficiency emerged as a consistent predisposing factor for CRAB bacteremia, given that secondary Abc complex bloodstream infection typically originates from respiratory tract infections (Kim et al., 2012). Hence, the utilization of ventilators has been found to contribute as a risk factor for the colonization and infection caused by Abc complex. The use of fiberoptic bronchoscopy is also a risk factor for CRAB bacteremia. The clinical application of bedside fiberoptic bronchoscopy is safe and convenient. Repeated sputum aspiration and alveolar lavage can dilute sputum and relieve airway obstruction, which is beneficial for the control of pulmonary infections and the regression of mucosal edema. However, there may be imperfect disinfection and the possibility of cross-infection. It is necessary to formulate and implement bunched intervention measures to strengthen the control of CRAB bloodstream infection, so as to reduce the risk of death of patients with CRAB bloodstream infection, pay attention to medical environment sanitation, strengthen the cleaning and disinfection of medical places, and equipment, strictly grasp the indications, implement the principle of aseptic operation, real-time dynamic evaluation of the necessity of various tubes, conditions allow early removal of tracheal intubation. Overall findings from drug resistance monitoring indicate that blood culture CRAB exhibits extensive resistance to commonly prescribed antibacterial medications while displaying high sensitivity toward polymyxin. Polymyxin, as a newly developed antibiotic, remains effective in treating CRAB infections and serves as the last resort option. Colistin is generally recommended for the treatment of CRAB unless severe renal insufficiency is present. These results can serve as valuable guidance when selecting appropriate antibiotics for patients infected with Abc complex.
- The detection of Abc complex bloodstream infection still relies on blood culture (Adrie et al., 2017), which takes a long time and has a low positive rate. Our hospital has implemented a system for promptly reporting critical values, wherein the microbiology laboratory is required to inform healthcare providers as soon as possible about the Gram-positive, Gram-negative, or candida nature of a positive blood culture result. This expedited reporting aids in guiding clinical decisions and minimizing delays in commencing antibiotic treatment. However, it typically takes 3–5 days to obtain a final outcome. Therefore, other methods should be used to identify and intervene early to improve the prognosis. In recent years, in-depth studies of inflammatory factors in the blood have led to a greater focus on the instructive significance of these inflammatory factors, which may have the value of predicting BSI for early clinical diagnosis or differential diagnosis. It has been reported in the literature that a large number of lymphocytes are apoptotic in patients with bloodstream infections, and that a continuous decrease in lymphocyte levels results in a dramatic decline in the body’s immune function. Therefore, low lymphocyte levels strongly correlate with the severity and prognosis of the patient. The count of leukocyte subsets in peripheral blood, especially an increase in neutrophils and a decrease in lymphocytes (i.e., neutrophil/lymphocyte ratio or NLR), can be used as an indicator of inflammatory status (Schroeder et al., 2001). Clinical neutrophil/lymphocyte ratio (NLR) can be obtained by peripheral blood routine, easy to use and inexpensive detection. Elevated NLR has demonstrated its usefulness in assessing the prognosis of individuals suffering from cardiovascular disorders (Durmus et al., 2015; Wada et al., 2017), sepsis (Riché et al., 2015; Hwang et al., 2017), cancer (Yodying et al., 2016) and various critical illnesses (Salciccioli et al., 2015). We observed a significant increase in NLR levels at 24, 48, and 72 h following Abc complex bloodstream infection among the non-surviving group compared to the surviving group (*p* < 0.05). The ROC curve showed that NLR had the highest AUC value of 0.727 at 48 h for predicting BSI prognosis. Additionally, an NLR > 20.8 at 48 h was identified as the critical threshold for predicting day 28 mortality, with a sensitivity of 57.14% and specificity of 87.88%. Meanwhile, the NLR levels at 24, 48, and 72 h following bloodstream infection were significantly elevated in the CRAB group compared to the CSAB group (p < 0.05). The ROC curve analysis demonstrated that at 72 h, the NLR exhibited a maximum AUC value of 0.700 for predicting drug resistance. Using an NLR threshold of >19.76 at 72 h as a critical point yielded a sensitivity of 47.83% and specificity of 83.78% for predicting drug resistance. The findings of this study suggest that the NLR measured 24 h after diagnosing Abc complex bloodstream infection lacks predictive value for both the patients’ 28-day prognosis and drug resistance. However, the NLR measured at 48 and 72 h exhibits some clinical significance in predicting both the patients’ 28-day prognosis and drug resistance, albeit with a relatively modest AUC. Our study implies that although NLR can be utilized to predict the prognosis and drug resistance in patients with Abc complex bloodstream infection, its predictive accuracy is limited; therefore, caution should be exercised when applying it to clinical diagnosis and treatment.

The SOFA score and APACHE II score are two commonly employed scoring systems utilized in the evaluation of critically ill patients. These scoring methods effectively assess the prognosis of critically ill patients, leading to their widespread usage (Qiao et al., 2012; Adam et al., 2013). The SOFA score is recommended as a clinical diagnostic criterion for sepsis and can be applied to determine the prognosis of ICU patients suspected of having an infection (AUC = 0.740) (Singer et al., 2016). APACHE II score is one of the most widely used methods to classify and evaluate the prognosis of critically ill patients and can evaluate the severity of the disease, the prognosis of patients, and the risk of death (Braber and van Zanten, 2010; Namendys-Silva et al., 2010); furthermore, the reliability and practicability have been widely recognized. With the increase in the APACHE II score, the nosocomial infection rate and ICU patient mortality have an increasing trend (Stevens et al., 2012). Godinjak et al. (2016) demonstrated that the APACHE II score exhibits significant discriminative ability in predicting sepsis prognosis, thereby highlighting its potential utility as a valuable prognostic tool. The study of Li and Li (2015) showed that APACHE II and SOFA scores had the same predictive ability for the prognosis of patients with sepsis. In this study, we conducted a comprehensive dynamic monitoring of both the SOFA score and APACHE II score, surpassing the depth achieved in our previous studies. The non-surviving group exhibited significantly elevated SOFA scores and APACHE II scores at each time point compared to the surviving group. Notably, a higher proportion of ICU hospitalization was observed in the non-survival group due to factors such as the severity of the underlying disease, high APACHE II score, and SOFA score, involvement of multiple organs, prolonged hospital stay, extensive use of broad-spectrum antibacterial medications, and various invasive procedures. These findings highlight important clinical implications for assessing disease progression and prognosis in critically ill patients (Molina et al., 2010). Multivariate logistic regression analysis revealed that the 24-h SOFA and APACHE II scores, as well as the APACHE II score at 72 h after diagnosis of Abc complex bloodstream infection, were independent risk factors for predicting the prognosis within 28 days (all *p* < 0.05). The ROC curve analysis demonstrated strong reliability in mortality prediction within 28 days among bacteremia patients with an AUC of 0.908 for the 24-h SOFA score and an AUC of 0.920 for the 72-h APACHE II score. Specifically, our findings indicated that a SOFA score exceeding 7 and an APACHE II score surpassing 23 were associated with an increased likelihood of death within this timeframe. Notably, the SOFA score exhibited exceptional prognostic value for individuals infected with Abc complex bloodstream infection by demonstrating remarkable sensitivity and specificity. Furthermore, there was no significant difference in the areas under the ROC curve at various time points following infection. There are many evaluation metrics for APACHE II scores, and the collection of clinical data is complicated. Ferreira et al. (2001) found that employing sequential SOFA scores offers a more efficient depiction of disease progression. Nevertheless, the computation process for such assessments can be quite time-consuming. We suggest utilizing the 24-h SOFA score as a prognostic indicator for patients diagnosed with bloodstream infection caused by Abc complex. From a clinical perspective, adopting a single scoring system proves to be more convenient and feasible. In this study, although the patients in the CRAB group exhibited significantly higher SOFA scores and APACHE II scores compared to those in the CSAB group on the day of bloodstream infection, as well as at 48 h and 72 h after infection (all p < 0.05), the discriminatory power of these scores, as indicated by the area under ROC curve at each time point, was limited. The ability to predict resistance in patients with Abc complex bloodstream infection was lower. Multivariate logistic regression analysis showed that admission to the ICU was the only independent risk factor for CRAB. Therefore, additional indicators and further studies are necessary to predict resistance to the Abc complex.

Limitations of this study include a small sample size and potential confounding factors. The laboratory of our hospital was unable to perform broth microdilution or gradient tests for carbapenems. The VITEK-2 Compact automated system possesses certain limitations in terms of antibiotic susceptibility testing, despite its ability to rapidly identify and test the susceptibility of numerous common bacteria using pre-prepared reagent cards. Future research should involve multiple institutions and diverse geographical areas to assess the effectiveness of scoring systems in predicting drug resistance in Abc complex infections.

## Conclusion

The severity of Abc complex bacteremia patients and the 28-day prognosis can be evaluated using both the SOFA score and APACHE II score. In terms of practicality, the SOFA score may offer more advantages than the APACHE II score for clinicians in predicting mortality rates due to its simpler and more convenient scoring system. Patients with CRAB bacteremia or sepsis exhibit significantly reduced survival time. Although NLR holds some clinical value in predicting 28-day mortality in Abc complex bloodstream infection patients, its predictive efficacy is limited. The early prediction of CRAB occurrence cannot be achieved through the utilization of SOFA score, APACHE II score, or NLR.

## Data availability statement

The original contributions presented in the study are included in the article/supplementary material, further inquiries can be directed to the corresponding authors.

## Ethics statement

The studies involving humans were approved by hospital's Ethics Committee (No. IRB2022-WZ-077). The studies were conducted in accordance with the local legislation and institutional requirements. Written informed consent for participation was not required from the participants or the participants' legal guardians/next of kin because a single-center retrospective study. Written informed consent was not obtained from the individual(s) for the publication of any potentially identifiable images or data included in this article because a single-center retrospective study.

## Author contributions

ZW: Data curation, Formal analysis, Writing – original draft, Writing – review & editing. LZ: Data curation, Formal analysis, Software, Writing – review & editing. JY: Conceptualization, Data curation, Formal analysis, Methodology, Writing – review & editing. XW: Data curation, Methodology, Project administration, Software, Writing – original draft. QL: Data curation, Formal analysis, Investigation, Software, Writing – original draft. YJ: Investigation, Project administration, Resources, Supervision, Validation, Visualization, Writing – original draft. JL: Investigation, Project administration, Software, Validation, Writing – review & editing. YC: Writing – review & editing. KX: Conceptualization, Funding acquisition, Resources, Supervision, Visualization, Writing – review & editing.
